# Radiotherapy interruption due to holidays adversely affects the survival of patients with nasopharyngeal carcinoma: a joint analysis based on large-scale retrospective data and clinical trials

**DOI:** 10.1186/s13014-022-02006-5

**Published:** 2022-02-19

**Authors:** Cheng Xu, Kai-Bin Yang, Rui-Jia Feng, Lei Chen, Xiao-Jing Du, Yan-Ping Mao, Wen-Fei Li, Qing Liu, Ying Sun, Jun Ma

**Affiliations:** 1 Department of Radiation Oncology, Sun Yat-Sen University Cancer Center, State Key Laboratory of Oncology in South China, Collaborative Innovation Center of Cancer Medicine, 651 Dongfeng Road East, Guangzhou, 510060 People’s Republic of China; 2 Zhongshan School of Medicine, Sun Yat-Sen University, Guangzhou, People’s Republic of China; 3 Department of Medical Statistics and Epidemiology, School of Public Health, Sun Yat-Sen University, Guangzhou, People’s Republic of China

**Keywords:** Nasopharyngeal carcinoma, Radiotherapy, Interruption, Prolongation, Holidays, Survival

## Abstract

**Background:**

The impact of radiotherapy interruption due to the Spring Festival holidays in China on the survival of patients with nasopharyngeal carcinoma (NPC) is unclear.

**Methods:**

Nontrial patients with locoregionally advanced NPC receiving radiotherapy plus induction chemotherapy (IC) and/or concurrent chemotherapy (CC) were included (*N* = 5035) and divided into two groups based on the Spring Festival-induced radiotherapy interruption. Kaplan–Meier curves for overall survival (OS) and failure-free survival (FFS) were compared between rival groups. Impact of the timing of radiotherapy interruption (during or outside the Spring Festival) on survival was investigated in a propensity score-matched dataset. We adopted ordination correspondence analysis to determine the cut-off of radiotherapy prolongation for prognostic prediction, and accordingly performed subgroup analysis based on delayed days and chemotherapy details. Individual patient data of three phase III NPC trials (NCT00677118, NCT01245959, NCT01872962) were used for validation (*N* = 1465).

**Results:**

Radiotherapy interruption was most frequently observed between December to January of the following year. Significantly lower OS and FFS were associated with the Spring Festival-induced interruption of radiotherapy (*P* = 0.009 and 0.033, respectively), but not that interruption of IC. In two matched comparison groups, the timing of radiotherapy interruption during the Spring Festival was more likely to lead to a decrease in FFS than outside the Spring Festival (*P* = 0.046), which was not observed in the validation using clinical trial data or in the subgroup analysis based on the 5-day delayed time. The absence of CC and the accumulated dose of cisplatin < 200 mg were related to the negative influences of the Spring Festival-induced radiotherapy interruption on FFS (*P* = 0.002) and OS (*P* = 0.010), respectively.

**Conclusions:**

The poor survival of patients with NPC is associated with the Spring Festival-induced interruption of radiotherapy. We recommend that these patients receive adequate doses of cisplatin concurrently with radiotherapy.

**Supplementary Information:**

The online version contains supplementary material available at 10.1186/s13014-022-02006-5.

## Introduction

Nasopharyngeal carcinoma (NPC) is a specific type of head and neck squamous cell carcinoma (HNSCC) prevalent in Southeast Asia. It is characterized by high radiosensitivity and a deep-seated anatomical location [1]. Radical radiotherapy (RT) is first-line therapy for early NPC, and also the cornerstone of multidisciplinary treatment for patients with locoregionally advanced NPC [2]. The advent of intensity-modulated radiotherapy (IMRT) has been reported to improve the 2-year locoregional control rate to 92.6%, in that this technique can deliver an adequate dose to the primary tumor while sparing surrounding normal tissues [3]. According to the standards and guidelines generated by the UK Royal College of Radiologists, patients with rapidly growing tumors (e.g., NPC) are classified as “Category One”, and their management is strongly dependent on the integrity and fidelity of RT implementation [4]. Therefore, the problems related to the quality of RT, such as the RT interruption, should be addressed thoroughly.

Scholars have raised concerns that uncompensated interruption of radical treatment occurs in over 30% patients with HNSCC [5, 6]. Thus, precise RT in NPC demands that its conventional fractionation and planned schedule should not be interrupted so that the best prognosis can be achieved [7]. Length of delayed time (“prolongation”) is the first critical aspect of treatment interruption, and also an indicator to measure its degree of severity [7, 8]. Evidence suggests that intervals of more than 10 days are related to a 10–20% reduction in the five-year survival rate of patients with HNSCC [9]. By contrast, few studies have focused on the second critical aspect—the timing of treatment interruption—which has become an urgent clinical issue and not yet been resolved [4]. The implementation of RT necessitates consideration of both clinical parameters (e.g., radiation dose, target volume) and psychosocial components (e.g., negative life event) [10]. This requires researchers to place the timing of RT gap in a wider context to further investigate its impact on the prognosis of cancer patients under a realistic and psycho-oncologic background. Surveys have suggested that the most important cause inducing RT interruption is public holidays (39–46%), which far exceeds those for maintenance of RT machines, patient compliance, and adverse events of treatment [11, 12]. The Spring Festival is not only the traditional Chinese New Year for Sinophone populations, but also a popular holiday worldwide, especially in Asia regions, most of which are NPC endemic areas [13]. Studies have shown that large-scale festivals have a considerable impact on an individual’s mood, psychology, and health condition [14, 15].

Therefore, it is of great clinical importance to explore the current situation of the implementation and interruption of RT in NPC. Based on large-scale retrospective data and three phase III randomized controlled trials, this joint analysis aimed to clarify the impact of the interaction between RT interruption and the Spring Festival on the survival of patients with NPC, which is expected to provide robust medical evidence on radiation oncology, and help physicians better guide and arrange precise RT in clinical practice.

## Materials and methods

The Institutional Review Board of Sun Yat-Sen University Cancer Center approved this study. Original data were uploaded to the Research Data Deposit (http://www.researchdata.org.cn) under the code RDDA2021001970. A prospective protocol was created in advance and registered at ClinicalTrial.gov (NCT04108338).

### Data sources and study design

A flowchart depicting the study design and inclusion/exclusion criteria is shown as Additional file 1: Figure S1. We included 5035 eligible cases from a NPC-specific dataset (*N* = 10,129) using a big-data intelligence platform (YiduCloud Technology, Beijing, China). All patients were diagnosed with newly diagnosed, non-disseminated, pathologically confirmed locoregionally advanced NPC, and receiving radical IMRT plus ≥ 2 cycles of induction chemotherapy (IC) and/or concurrent chemotherapy. In addition, 1465 trial participants with NPC from three phase III randomized controlled trials (NCT00677118, NCT01245959, and NCT01872962) were screened out as the dataset for validation.

### Pre-treatment workup

Clinical staging was guided by the 8th edition of the American Joint Committee on Cancer/Union for International Cancer Control manual. All patients underwent the following examinations within the 2 weeks before treatment initiation: complete medical history, physical examination, hematology and biochemistry profiles, plasma Epstein–Barr virus (EBV) DNA titer, nasopharyngeal fiberoptic endoscopy, neck and nasopharyngeal magnetic resonance imaging, chest radiography or computed tomography (CT), abdominal ultrasound and skeletal scintigraphy. ^18^F-fluorodeoxyglucose positron emission tomography/CT was used to replace the latter three items for detection of possible metastases. A real-time quantitative polymerase-chain-reaction assay that targets the *Bam*HI-W region of the EBV genome was used to detect plasma EBV DNA [16].

### RT and chemotherapy

Based on expert consensus and reports on RT, all patients received radical IMRT to treat the nasopharyngeal and neck tumor volumes for the entire course [3]. Prescribed doses were administered in 28–33 fractions (38–45 days; one fraction daily) using the simultaneous integrated boost technique. The conventional fractionation of RT was implemented in five consecutive weekdays with a weekend break according to the planned schedule. Description of treatment is detailed in the Supporting information.

### Follow-up and endpoints

Follow-up was measured from the day of the diagnosis to the day of the final examination or death. Each patient attended a follow-up appointment at least every 3 months during the first 2 years, then every 6 months thereafter or until death. If a patient had suspected residual or recurrent local disease, a biopsy was required to confirm malignancy. The endpoints were overall survival (OS) and failure-free survival (FFS). OS was measured from the day of the diagnosis until death due to any cause or the latest known date alive; FFS, to failure, death from any cause or last follow-up visit, whichever occurred first.

### Statistical analysis

The date of the Spring Festival in the traditional lunar calendar is not fixed, so we screened out the patients whose time span of IC or RT covered the Spring Festival by querying the annual holiday dates between 2010 and 2016. A second calculation of delayed time was performed and compared with the routine RT schedule to further confirm that the RT interruption occurred during the Spring Festival. When the time period of RT interruption overlapped with the holiday, it was defined as “during the Spring Festival”; otherwise, it was defined as “outside the Spring Festival”. Descriptive statistics provided as continuous variables were converted into categorical variables according to the interquartile range (IQR; age) and cutoff points reported in clinical examination (serum lactate dehydrogenase [LDH], C-reactive protein [CRP], and anemia) or in previous studies (EBV DNA) [17]. Actuarial survival curves were estimated using the Kaplan–Meier method and compared using log-rank tests [18]. Hazard ratios and 95% confidence intervals were measured using Cox regression to quantify the effect of potential confounders on survival outcomes.

To minimize the influence of observed confounders on a selection bias, a 1-to-1 propensity score matching (PSM) method without replacement was performed for the comparison between rival groups using the nearest-neighbor method with a stringent caliper of 0.05 [19]. Multivariate ordination correspondence analysis via log-binomial regression was used to explore the association between categorical variables and prognostic outcomes, as well as to determine the cutoff value of RT prolongation for prognostic prediction [20]. Accordingly, we performed subgroup analysis based on the number of days RT was delayed and chemotherapy details. Data visualization of histograms and curves were developed using Tableau Desktop, 2018 (Tableau Software, Seattle, WA, USA). Statistical analyses were carried out using SPSS 24.0 (IBM, Armonk, NY, USA) or the *RMS* and *MASS* packages in R v3.3.2 (www.r-project.org/). All *P* values were two-sided, with significance defined as a *P* < 0.05.

## Results

### Baseline characteristics and general trend of RT interruption

The median age of the entire cohort was 44 (IQR = 38–52) years. A male-to-female ratio of 2.75-to-1 and a predominant (97.7%) histology type of non-keratinizing undifferentiated NPC (World Health Organization [WHO] type III) were documented. More than half (58.8%) of patients received multimodality treatment based on IC followed by RT, and 41.2% of them were treated by concurrent chemotherapy plus RT. The Spring Festival-induced interruption of IC and RT accounted for 14.2% and 11.2% of cases, respectively. Univariate Cox analysis indicated gender, histological type, T category, N category, RT interruption during the Spring Festival, cigarette smoking, as well as levels of EBV DNA, LDH, and CRP, to have significant effects on OS and FFS (Table 1).

**Table 1:** Baseline characteristics and univariate analysis in 5035 patients with NPC

Characteristic	Entire cohort no. (%)^a^	Univariate Cox analysis			
		5-year OS		5-year FFS	
		HR (95% CI)	*P*	HR (95% CI)	*P*
*Age, years*					
< 37	1175 (23.3)	Reference		Reference	
38–43	1141 (22.7)	1.30 (1.01–1.67)	0.043	1.11 (0.93–1.33)	0.247
44–51	1380 (27.4)	1.35 (1.06–1.71)	0.016	1.06 (0.89–1.27)	0.483
≥ 52	1339 (26.6)	2.00 (1.59–2.51)	< 0.001	1.17 (0.98–1.39)	0.079
*Gender*					
Male	3697 (73.4)	Reference		Reference	
Female	1338 (26.6)	0.63 (0.57–0.84)	< 0.001	0.74 (0.64–0.86)	< 0.001
*Histological type*					
WHO type I–II	116 (2.3)	Reference		Reference	
WHO type III	4919 (97.7)	0.55 (0.37–0.80)	0.002	0.51 (0.37–0.68)	< 0.001
*T category (8th edition)*					
T1	263 (5.2)	Reference		Reference	
T2	364 (7.2)	1.26 (0.80–2.00)	0.322	1.20 (0.84–1.71)	0.327
T3	3049 (60.6)	1.02 (0.70–1.48)	0.936	1.01 (0.75–1.35)	0.973
T4	1359 (27.0)	1.66 (1.13–2.44)	0.010	1.51 (1.12–2.04)	0.007
*N category (8th edition)*					
N0	456 (9.1)	Reference		Reference	
N1	2335 (46.4)	1.67 (1.14–2.46)	0.009	1.59 (1.19–2.11)	0.002
N2	1455 (28.9)	2.50 (1.70–3.69)	< 0.001	2.23 (1.67–2.98)	< 0.001
N3	789 (15.7)	3.57 (2.41–5.31)	< 0.001	3.00 (2.22–4.03)	< 0.001
*Treatment*^*b*^					
IC + CCRT	2420 (48.0)	–	–	–	–
IC + RT	543 (10.8)	–	–	–	–
CCRT	2072 (41.2)	–	–	–	–
*IC interruption during the Spring Festival*					
No	2542 (85.8)	Reference		Reference	
Yes	421 (14.2)	1.06 (0.81–1.38)	0.692	0.94 (0.75–1.17)	0.565
*RT interruption during the Spring Festival*					
No	4473 (88.8)	Reference		Reference	
Yes	562 (11.2)	1.34 (1.08–1.67)	0.009	1.22 (1.02–1.46)	0.033
*EBV DNA titer, copies/mL*					
< 2000	2197 (43.6)	Reference		Reference	
≥ 2000	2838 (56.4)	2.08 (1.75–2.47)	< 0.001	2.03 (1.78–2.32)	< 0.001
*LDH, U/L*					
< 250	4651 (92.4)	Reference		Reference	
≥ 250	384 (7.6)	2.07 (1.66–2.59)	< 0.001	1.67 (1.38–2.03)	< 0.001
*CRP, mg/L*					
≤ 3.00	3387 (67.3)	Reference		Reference	
> 3.00	1648 (32.7)	1.60 (1.37–1.86)	< 0.001	1.31 (1.16–1.48)	< 0.001
*Anemia*					
No	4817 (95.7)	Reference		Reference	
Yes	218 (4.3)	1.44 (1.04–2.00)	0.028	1.15 (0.87–1.53)	0.320
*Family history of cancer*					
No	3788 (75.2)	Reference		Reference	
Yes	1247 (24.8)	0.92 (0.77–1.11)	0.376	1.01 (0.88–1.16)	0.883
*Cigarette smoking*					
No	3185 (63.3)	Reference		Reference	
Yes	1850 (36.7)	1.34 (1.15–1.57)	< 0.001	1.26 (1.12–1.42)	< 0.001
*Alcohol consumption*					
No	4330 (86.0)	Reference		Reference	
Yes	705 (14.0)	1.14 (0.92–1.41)	0.230	1.07 (0.90–1.27)	0.436
*Comorbidity*					
No	3936 (78.2)	Reference		Reference	
Yes	1099 (21.8)	1.08 (0.90–1.30)	0.403	1.04 (0.90–1.20)	0.633

NPC, nasopharyngeal carcinoma; no., number; OS, overall survival; FFS, failure-free survival; HR, hazard ratio; CI, confidence interval; WHO, World Health Organization; CCRT, concurrent chemoradiotherapy; IC, induction chemotherapy; RT, radiotherapy; EBV, Epstein-Barr virus; LDH, lactate dehydrogenase; CRP, C-reactive protein

^a^Percentages may not add up to 100 due to rounding up of values

^b^All chemoradiotherapy was based on intensity-modulated radiation therapy

**Fig. 1:**
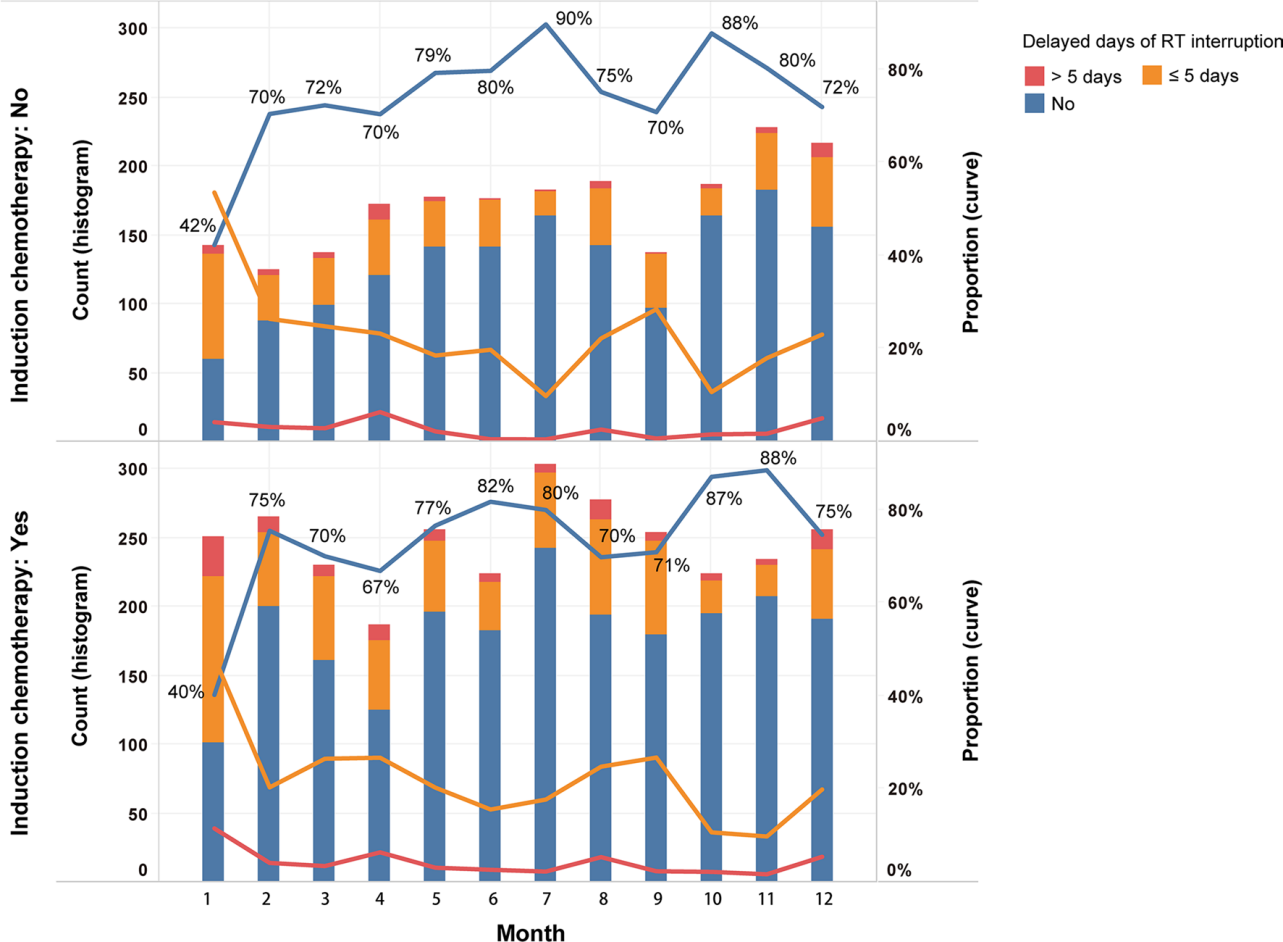
Counts and proportion per month of RT interruption according to delayed days. *RT* radiotherapy

The addition of IC reduced the relative uniformity of monthly RT counts. The highest proportion of RT interruption was between December to January of the following year, with up to 60% and 58% in the IC subgroup and non-IC subgroup, respectively. Only the patients having RT prolongation ≤ 5 days had the opposite evolving trend corresponding to the no interruption group (Fig. 1).

### Treatment interruption during the Spring Festival

**Fig. 2:**
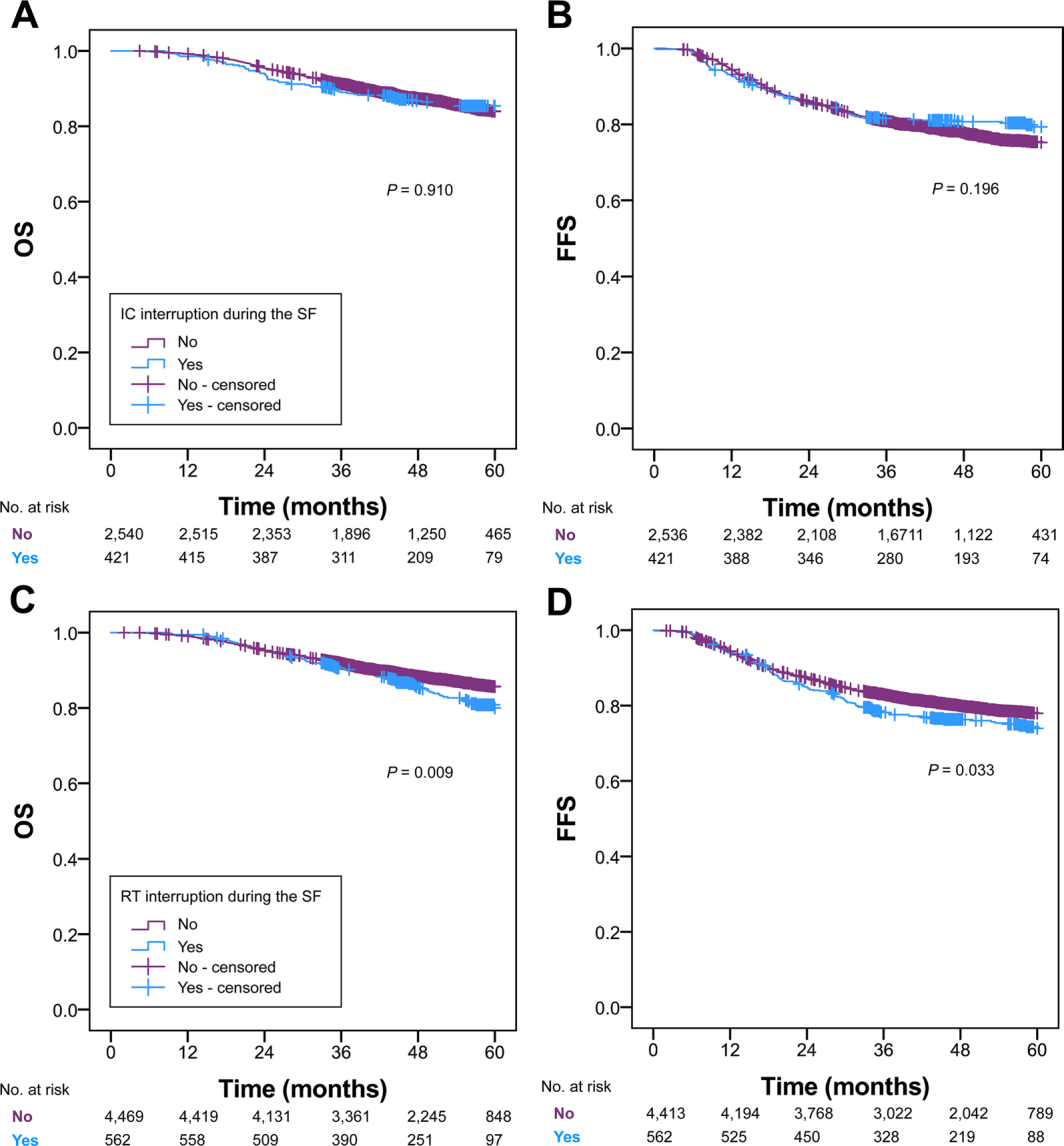
Kaplan–Meier survival curves for OS and FFS stratified by whether IC interruption (**a–b**) and RT interruption (**c–d**) occurred during the Spring Festival. *OS* overall survival, *FFS* failure-free survival, *IC* induction chemotherapy, *RT* radiotherapy, *SF* Spring Festival

The interruption of IC caused by the Spring Festival had non-significant influence on OS (*P* = 0.910) and FFS (*P* = 0.196) of patients with locoregionally advanced NPC (Fig. 2). Significantly lower survival outcomes were associated with the Spring Festival-induced RT interruption, with 5-year OS of 80.0% versus 85.9% (*P* = 0.009) and 5-year FFS of 74.0% versus 78.0% (*P* = 0.033).

### Impact of the timing and prolongation of RT interruption on survival

**Fig. 3:**
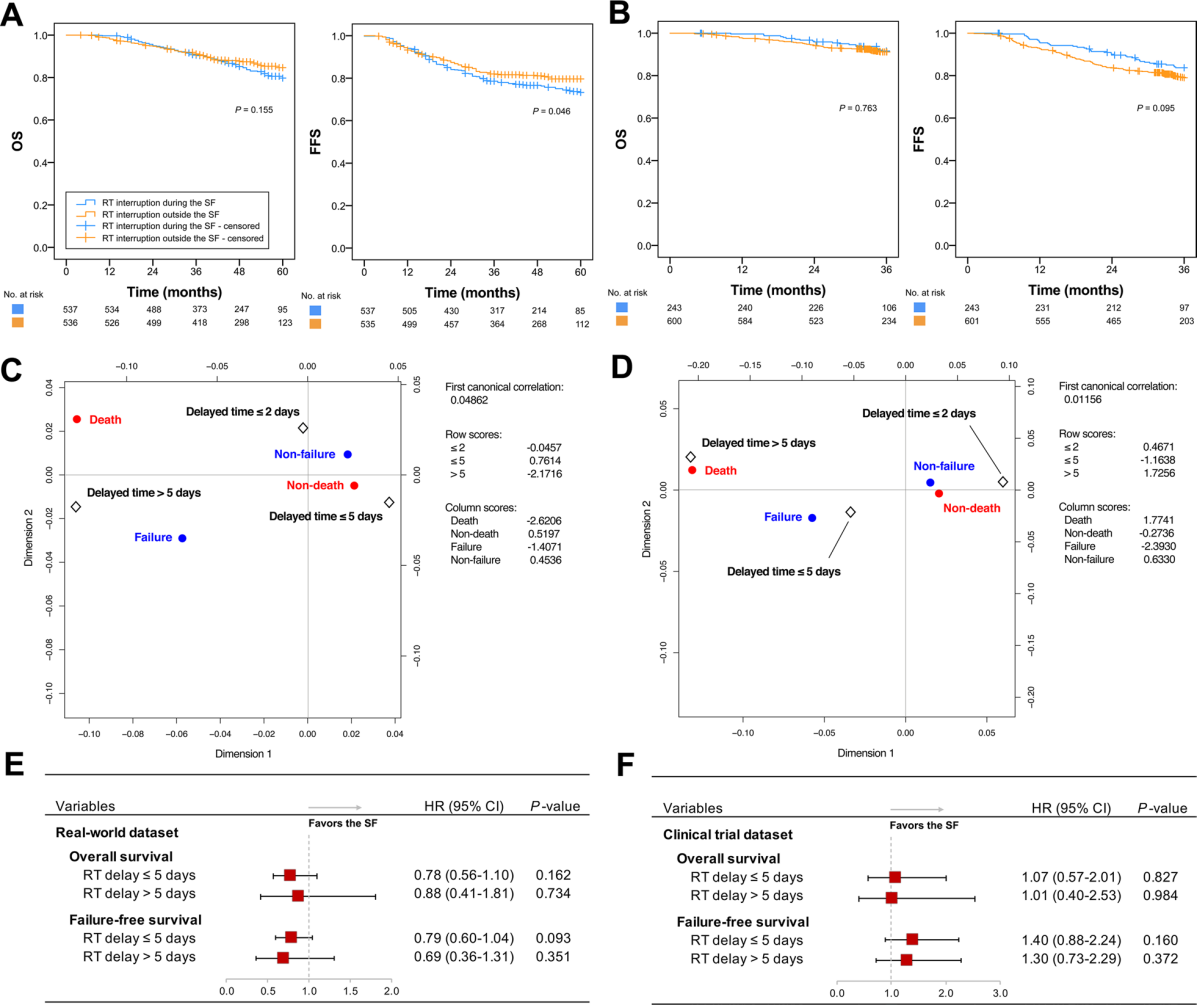
Analyses of the relationship between the timing (**a, b**) and prolongation (**c–f**) of RT interruption and the prognosis of NPC patients based on real-world and clinical trial datasets. Geometric biplots of the correspondence analyses were carried out based on the delayed days caused by RT interruption during (**c**) and outside (**d**) the Spring Festival. *NPC* nasopharyngeal carcinoma, *OS* overall survival, *FFS* failure-free survival, *RT* radiotherapy, *SF* Spring Festival, *HR* hazard ratio

The 1-to-1 PSM created two well-matched comparison groups of RT interruption during or outside the Spring Festival (*N* = 537 vs. 537; all *P *values ≥ 0.422) to investigate the timing of interruption on survival (Additional file 1: Table S1). The timing of RT interruption during the Spring Festival was more likely to lead to a decrease in FFS than that outside the Spring Festival (*P* = 0.046). Individual patient data of three phase III NPC trials showed non-significantly separated survival curves between the timing of RT interruption during versus outside the Spring Festival (244 vs. 602; all *P* values ≥ 0.095) (Fig. 3a, b).

The contribution of treatment failure and death was accounted for mainly by a delayed time ≤ 5 days in RT interruption during (Fig. 3c) and outside (Fig. 3d) the Spring Festival, with a close distance and linkage between the scattered points in a different sign of the row score from other time nodes (0.7614 and − 1.1638, respectively). Figure 3e, f suggest that the 5-day prolongation caused by Spring Festival-induced RT interruption did not affect the survival of NPC patients significantly. Equivalent OS and FFS between the RT interruption during and outside the Spring Festival was, in general, observed in real-world and clinical trial settings, whereas the two settings had opposite trends in the impact of RT interruption on survival (Fig. 3e, f).

### Subgroup analysis

**Fig. 4:**
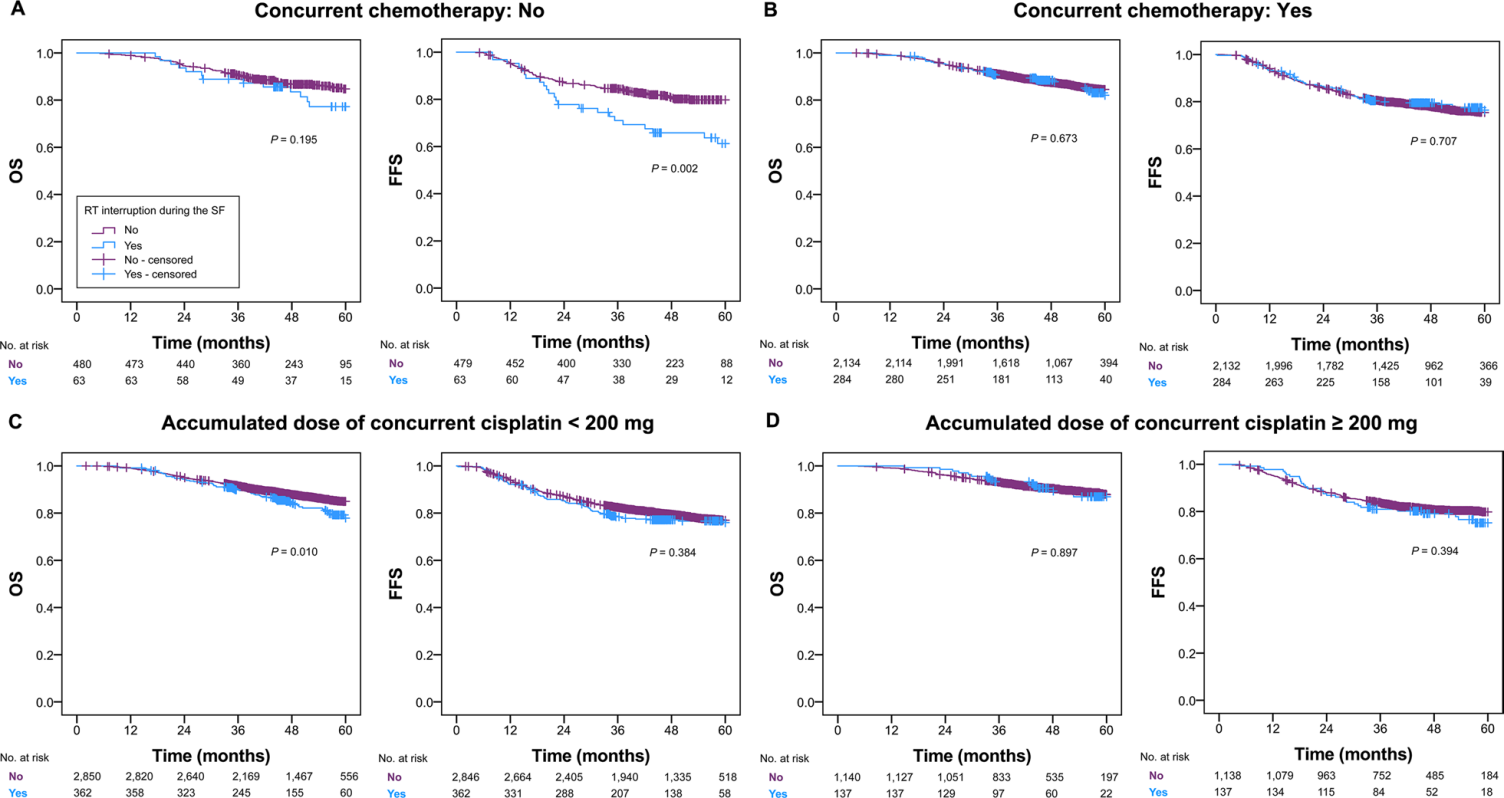
Kaplan–Meier survival curves stratified by the timing of RT interruption in the subgroup of concurrent chemotherapy (**a, b**) and accumulated dose of concurrent cisplatin (**c, d**). OS, overall survival; FFS, failure-free survival; RT, radiotherapy; SF, Spring Festival

The absence of concurrent chemotherapy and an accumulated dose of cisplatin < 200 mg were related to the adverse influences of the Spring Festival-induced RT interruption on FFS (*P* = 0.002) and OS (*P* = 0.010), respectively (Fig. 4). Moreover, the adverse effect of RT interruption during the Spring Festival on OS and/or FFS was validated in the subgroups of female gender, WHO type III, T3 category, EBV DNA < 2000 copies/mL, LDH < 250 U/L, and CRP > 3.00 mg/L (all *P* values ≤ 0.048) (Table 2).

**Table 2:** Impact of RT interruption during the Spring Festival on OS and FFS based on validated predictive factors

Characteristics	5-year OS		5-year FFS	
	HR (95% CI)^a^	*P*	HR (95% CI)^a^	*P*
*Gender*				
Male	1.17 (0.91–1.51)	0.229	1.14 (0.93–1.40)	0.206
Female	2.07 (1.35–3.18)	0.001	1.48 (1.03–2.13)	0.035
*Histological type*				
WHO type I–II	0.68 (0.16–2.85)	0.595	1.27 (0.50–3.23)	0.610
WHO type III	1.37 (1.10–1.71)	0.005	1.22 (1.01–1.46)	0.035
*T category (8th edition)*				
T1	1.33 (0.40–4.40)	0.639	1.07 (0.38–2.97)	0.900
T2	0.56 (0.17–1.78)	0.323	0.86 (0.40–1.87)	0.704
T3	1.47 (1.09–2.00)	0.012	1.26 (0.98–1.62)	0.068
T4	1.23 (0.87–1.73)	0.250	1.15 (0.86–1.53)	0.345
*N category (8th edition)*				
N0	1.98 (0.75–5.18)	0.165	1.46 (0.66–3.23)	0.353
N1	1.40 (0.99–1.98)	0.058	1.22 (0.92–1.61)	0.160
N2	1.26 (0.85–1.89)	0.254	1.23 (0.89–1.71)	0.211
N3	1.23 (0.79–1.91)	0.367	1.14 (0.78–1.66)	0.498
*EBV DNA titer, copies/mL*				
< 2000	1.53 (1.02–2.29)	0.039	1.31 (0.95–1.82)	0.102
≥ 2000	1.26 (0.97–1.63)	0.087	1.17 (0.94–1.45)	0.153
*LDH, U/L*				
< 250	1.37 (1.08–1.73)	0.009	1.27 (1.05–1.53)	0.013
≥ 250	1.10 (0.60–2.02)	0.754	0.78 (0.43–1.41)	0.408
*CRP, mg/L*				
≤ 3.00	1.21 (0.89–1.64)	0.235	1.13 (0.89–1.43)	0.320
> 3.00	1.46 (1.07–1.99)	0.019	1.32 (1.00–1.73)	0.048
*Cigarette smoking*				
No	1.33 (0.99–1.77)	0.058	1.19 (0.94–1.51)	0.138
Yes	1.37 (0.98–1.92)	0.064	1.25 (0.95–1.66)	0.111

OS, overall survival; FFS, failure-free survival; HR, hazard ratio; CI, confidence interval; WHO, World Health Organization; CCRT, concurrent chemoradiotherapy; IC, induction chemotherapy; RT, radiotherapy; EBV, Epstein–Barr virus; LDH, lactate dehydrogenase; CRP, C-reactive protein

^a^HR > 1 indicates an increase in the negative effect of RT interruption during the Spring Festival on survival outcomes

## Discussion

Treatment interruption is very common and may be caused by exacerbation of health conditions, comorbidities, chemotherapy complications, and availability of radiation facilities. Researchers have conducted a lot of studies on the prolongation of treatment due to unplanned interruptions, and it is generally acknowledged that the prolonged treatment delay has a detrimental effect on the prognosis of cancer patients [7, 21]. In addition, scholars have focused on the long waiting time between the diagnosis and treatment in different cancer types, which is obviously affected by socioeconomic factors, such as low-efficiency healthcare process, limited medical resources, and absence of psychosocial support [22, 23]. With regard to the problem of the timing of RT interruption, Kwong et al. simply explored the position of RT interruption in the entire treatment course and found that a gap at the beginning or towards the end of treatment did not significantly alter outcomes [24]. Skladowski et al. reported that patients with supraglottic larynx cancer suffering RT interruption initiated before day 19 after the start of therapy yielded lower local tumor control than those who did not have a gap in treatment [25]. However, no studies have reported the interaction between the timing of RT interruption and psycho-oncologic events in cancer, which is possibly associated between a worse prognosis in radiation oncology clinics [26]. Our findings suggested that worse survival of patients with NPC was related to the Spring Festival-induced interruption of RT. Adequate doses of concurrent cisplatin may help to reduce this adverse influence.

When interpreting our findings, three factors from the perspectives of radiobiology, psychology, and environment should be considered. First, most staff and linear accelerators were off-work with an average period of 5.28 days during the Spring Festival [27]. RT interruption for nearly 1 full work week and the decrease in utilization of radiation services may compromise the therapeutic benefits of patients because of reduced biological efficacy [28]. Second, stress often takes on a different character during a holiday than at other times of the year, which may be caused mainly by hectic preparations (67%), financial burden (62%), and the hype of season (53%) [29]. Evidence has suggested psychological impairments can lead to substantial physical problems, with increased morbidity and mortality [30, 31]. Besides, many individuals may engage in “comfort eating”, alcohol consumption, and sedentary activities in a holiday to cope with their stress levels, and these unhealthy behaviors are closely related to deterioration of tumor conditions [32]. Third, Phillips et al. reported that the top three disease groups with a spiking mortality during Christmas and New Year holidays are circulatory diseases, neoplasms, and respiratory diseases [33]. This observation is in accordance with our findings that the Spring Festival may be a risk factor for death and treatment failure for patients with NPC. One possible reason is that these holiday seasons are located in the winter months, which happens to be a special period associated with a higher incidence of diseases [15].

The association between holidays and unfavorable health-related outcomes exists in different nationalities, cultures, and ethnicities, so there must be an intrinsic cause of the “holiday effect” [34, 35]. RT efficacy in clinical practise is deemed to be determined based on the information related to both disease-related factors and psychosocial components, which usually interact with each other constantly in an unpredictable manner [35]. This is a hitherto unexplored area in NPC and has never been assessed. Patients with NPC who suffered RT interruption during the Spring Festival period indeed differed from those receiving scheduled conventional fractionation of RT outside the Spring Festival, starting from their baseline characteristics of clinical complexity (i.e., gender, histological type, T category, EBV DNA titer, LDH, and CRP). A past research had suggested that women are more vulnerable to increased stress around a holiday season than men (44% vs. 31%) because they shoulder the majority of the family burden and have particular stress due to the time constraints required to prepare the holiday celebrations [29]. According to one robust systematic review, the highest median prevalence of decreased psychosocial function in the entire course of RT was during RT (36%) rather than before or after RT (20–25%) [24]. Moreover, 17 predictors of psychosocial decline were identified and grouped into five categories: female gender, time point during RT, physical symptoms, chemotherapy receipt, and younger age [24]. In this joint analysis based on large-scale retrospective data and clinical trials, we not only explored the specific time point of RT interruption during the Spring Festival and its impact on the survival of NPC patients, but also validated the three predictors—female gender, physical symptoms (e.g., heavy tumor burden as reflected by advanced T stage), chemotherapy reception (e.g., adequate dose of concurrent cisplatin)—that may be candidate components of the clinical complexity of a NPC population and the critical factors used to measure their psycho-oncologic status qualitatively. One possible reason for the failure of the analysis based on the clinical trial dataset to validate the results of real-word data is the differences in RT quality assurance between trial and non-trial patients. Mounting evidence suggests that centers participating in clinical trials undergoing favorable treatment quality, centers with high case volume, and academic centers are more likely to yield superior outcomes compared with their counterparts [36, 37].

This study has some weaknesses that should be mentioned. First, providing solid evidence in terms of causation between the observed high risk of poor survival outcomes of patients with NPC and RT interruption during the Spring Festival was difficult. Psychosocial status is affected by multifaceted and multidimensional variables, so we discussed only some possible reasons and skepticisms. Nonetheless, the aforementioned predictors can explain our findings only partially. In fact, dealing with the adverse impact of the Spring Festival on survival is one of the most challenging, yet still unmet, requirements of the RT implementation in NPC. Second, the NPC population from endemic areas (e.g., Southern China) was a suitable targeted group for research on the Spring Festival and survival. However, this feature will, to some extent, limit the generalizability of our results, which were from an endemic single center. Third, the findings shown in Fig. 1 also indicated the second highest proportion of RT interruption was September-to-October. This period exactly overlaps with the China National Day, which has a same 7-day holiday as the Spring Festival. We did not conduct further investigation of this topic because the statistical significance of our results may have been reduced if multiple independent hypotheses were tested simultaneously on the same dataset [38]. Therefore, whether there are differences in the impact of different festivals on the prognosis of patients with NPC is not known.

## Supplementary Information

**Additional file 1: Figure S1.** Flowchart of the study design and identification of eligible patients. **Table S1.** Patients with NPC suffering RT interruption during or outside the Spring Festival in the matched cohort from the real-world dataset.

## Data Availability

The data that support the findings of this study are available from the Research Data Deposit (http://www.researchdata.org.cn) but restrictions apply to the availability of these data, which were used under license for the current study (RDDA2021001970), and so are not publicly available. Data are, however, available from the authors upon reasonable request.

## Abbreviations

NPC: Nasopharyngeal carcinoma
IC: Induction chemotherapy
CC: Concurrent chemotherapy
CRP: C-reactive protein
CT: Computed tomography
EBV: Epstein-Barr virus
FFS: Failure-free survival
HNSCC: Head and neck squamous cell carcinoma
IMRT: Intensity-modulated radiotherapy
IQR: Interquartile range
LDH: Serum lactate dehydrogenase
OS: Overall survival
RT: Radiotherapy
PSM: Propensity score matching
SF: Spring Festival
WHO: World Health Organization
