# Supplementary material for: Radiotherapy interruption due to holidays adversely affects the survival of patients with nasopharyngeal carcinoma: a joint analysis based on large-scale retrospective data and clinical trials
Source: Radiat Oncol. 2022 Feb 19;17:36. doi: 10.1186/s13014-022-02006-5 (PMC8858542; doi:10.1186/s13014-022-02006-5)
Supplement: Supplementary file 1 — Additional file 1: Figure S1. Flowchart of the study design and identification of eligible patients. Table S1. Patients with NPC suffering RT interruption during or outside the Spring Festival in the matched cohort from the real-world dataset. [file 13014_2022_2006_MOESM1_ESM.docx]

**Methods**

***Radiotherapy and chemotherapy***

Based experts’ consensus and previous reports on RT, all patients will receive radical IMRT to treat the nasopharyngeal and neck tumor volumes for the entire course [1]. Patients were immobilized in the supine position using a head, neck, and shoulder thermoplastic mask. Intravenous contrast CT simulation is performed at 3 mm intervals from the head to 2 cm below the sternoclavicular joint using a CT simulator. Target volumes will be delineated slice-by-slice on treatment planning CT scans using an individualized delineation protocol in accordance with the International Commission on Radiation Units and Measurements reports 50 and 62 [2]. The planning target volumes (PTVs) and planning organs at risk volume (PRVs) will be generated by the addition of a 3 mm margin to both the delineated target volume and corresponding structures (such as the spinal cord, brainstem, and optic nerve pathway). The PTV of the high-risk clinical target volume (CTV1) will be extended 5–10 mm beyond the margin of the primary gross tumor volume (GTVnx) for potential microscopic spread, including the entire nasopharyngeal mucosa and 5 mm into the submucosal region. The PTV of the low-risk clinical target volume (CTV2) will be extended 5–10 mm beyond the margin of the CTV1, potentially involving regions and lymphatic regions, unless the CTV2 was adjacent to critical organs, e.g., brain stem and spinal cord, in which case the extension distance will be reduced to 3–5 mm. Prescribed doses were administered in 28–33 fractions (38–45 days; one fraction daily) using the simultaneous integrated boost technique as follows: 66–72 Gy to the planning target volume (PTV) of nasopharyngeal gross tumor volume (GTV), 64–70 Gy to the PTV of the GTV of the metastatic lymph nodes, 60–63/54–56 Gy to the PTV of the high-/low-risk clinical target volume. The split-course of RT was implemented in five consecutive weekdays with a weekend break according to the planned fractionation schedule. The optimization and evaluation of plan design is based on the standard dose constraint set of RTOG 0615 and 0225 [3, 4].

IC regimens consisted of cisplatin–5-fluorouracil (80 mg/m^2^ and 4,000 mg/m^2^, respectively), docetaxel–cisplatin (75 mg/m^2^ and 75 mg/m^2^, respectively), docetaxel–cisplatin–5-fluorouracil (60 mg/m^2^, 60 mg/m^2^, and 3,000 mg/m^2^, respectively), and gemcitabine-cisplatin (1000 mg/m^2^ on days 1 and 8, and 80 mg/m^2^ on day 1, respectively), every three weeks for more than two cycles. Concurrent chemotherapy regimens consisted of weekly (30–40 mg/m^2^) or three-weekly cisplatin (80–100 mg/m^2^) for more than two cycles.

**References**

1. Lai SZ, Li WF, Chen L, Luo W, Chen YY, Liu LZ, et al. How does intensity-modulated radiotherapy versus conventional two-dimensional radiotherapy influence the treatment results in nasopharyngeal carcinoma patients? *Int J Radiat Oncol Biol Phys* 2011;80(3):661-8.

2. Measurements ICoRUa. Report 62: Prescribing, recording and reporting pho-ton beam therapy (supplement to ICRU report 50). Bethesda, MD: ICRU 1999.

3. Lee N, Harris J, Garden AS, Straube W, Glisson B, Xia P, et al. Intensity-modulated radiation therapy with or without chemotherapy for nasopharyngeal carcinoma: radiation therapy oncology group phase II trial 0225. *J Clin Oncol* 2009;27(22):3684-90.

4. Lee NY, Zhang Q, Pfister DG, Kim J, Garden AS, Mechalakos J, et al. Addition of bevacizumab to standard chemoradiation for locoregionally advanced nasopharyngeal carcinoma (RTOG 0615): a phase 2 multi-institutional trial. *Lancet Oncol* 2012;13(2):172-80.

**
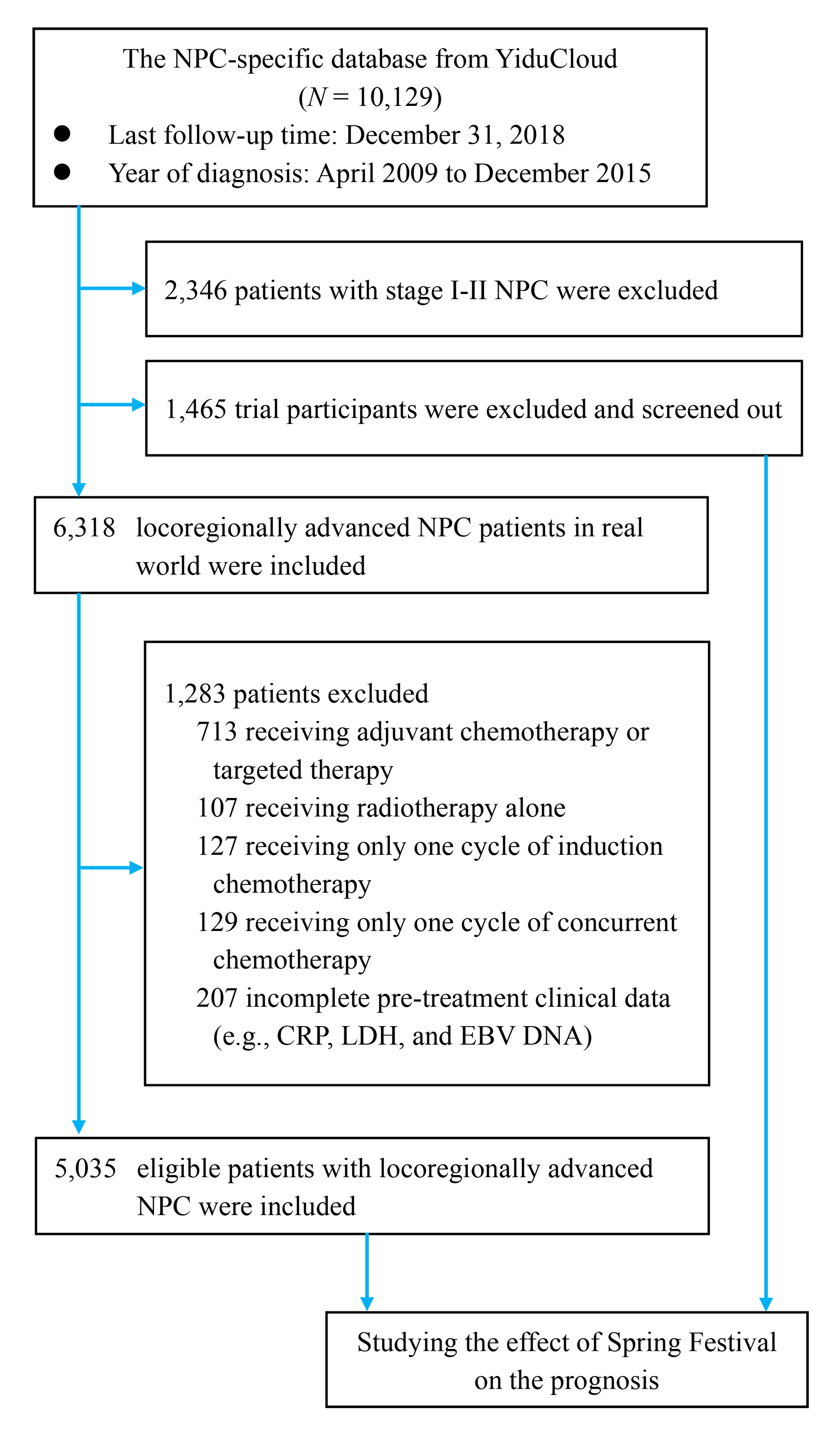
**

**Fig. S1.** Flowchart of the study design and identification of eligible patients. NPC, nasopharyngeal carcinoma; CRP, C-reactive protein; LDH, lactate dehydrogenase; EBV, Epstein–Barr virus.

**Table S1. Patients with NPC suffering RT interruption during or outside the Spring Festival in the matched cohort from the real-world dataset**

| Variables | RT interruption during the Spring Festival, n (%) | RT interruption outside the Spring Festival, n (%) | *P* |
| --- | --- | --- | --- |
| Sample size | 537 | 537 | - |
| Age, years |  |  | 0.792 |
| < 37 | 130 (24.2) | 133 (24.8) |  |
| 38-43 | 115 (21.4) | 117 (21.8) |  |
| 44-51 | 142 (26.4) | 128 (23.8) |  |
| ≥ 52 | 150 (27.9) | 159 (29.6) |  |
| Gender |  |  | 0.447 |
| Male | 401 (74.7) | 389 (72.4) |  |
| Female | 136 (25.3) | 148 (27.6) |  |
| Histological type |  |  | 1.000 |
| WHO type I-II | 9 (1.7) | 8 (1.5) |  |
| WHO type III | 528 (98.3) | 529 (98.5) |  |
| T category (8th edition) | |  | 0.813 |
| T1 | 21 (3.9) | 20 (3.7) |  |
| T2 | 32 (6.0) | 35 (6.5) |  |
| T3 | 302 (56.2) | 314 (27.2) |  |
| T4 | 182 (33.9) | 168 (31.3) |  |
| N category (8th edition) | |  | 0.848 |
| N0 | 43 (8.0) | 37 (6.9) |  |
| N1 | 268 (49.9) | 263 (49.0) |  |
| N2 | 137 (25.5) | 146 (27.2) |  |
| N3 | 89 (16.6) | 91 (16.9) |  |
| EBV DNA titer, copies/mL | |  | 0.422 |
| < 2,000 | 233 (43.4) | 219 (40.8) |  |
| ≥ 2,000 | 304 (56.6) | 318 (59.2) |  |
| Treatment |  |  | 0.493 |
| IC + CCRT | 267 (49.7) | 248 (46.2) |  |
| IC + RT | 61 (63.6) | 68 (12.7) |  |
| CCRT | 209 (38.9) | 221 (41.2) |  |
| LDH, U/L |  |  | 1.000 |
| < 250 | 495 (92.2) | 496 (92.4) |  |
| ≥ 250 | 42 (7.8) | 41 (7.6) |  |
| CRP, mg/L |  |  | 0.847 |
| ≤ 3.00 | 350 (65.2) | 354 (65.9) |  |
| > 3.00 | 187 (34.8) | 183 (34.1) |  |
| Anemia |  |  | 0.887 |
| No | 510 (95.0) | 512 (95.3) |  |
| Yes | 27 (5.0) | 25 (4.7) |  |
| Cigarette smoking |  |  | 0.850 |
| No | 340 (63.3) | 336 (62.6) |  |
| Yes | 197 (36.7) | 201 (37.4) |  |

NPC, nasopharyngeal carcinoma; RT, radiotherapy; WHO, World Health Organization; EBV, Epstein–Barr virus; DNA, deoxyribonucleic acid; IC, induction chemotherapy; CCRT, concurrent chemoradiotherapy; LDH, lactate dehydrogenase level; CRP, C-reactive protein.
